# Task Offloading and Resource Allocation Strategy in Non-Terrestrial Networks for Continuous Distributed Task Scenarios

**DOI:** 10.3390/s25196195

**Published:** 2025-10-06

**Authors:** Yueming Qi, Yu Du, Yijun Guo, Jianjun Hao

**Affiliations:** 1 Beijing Key Laboratory of Network System Architecture and Convergence, Beijing University of Posts and Telecommunications, Beijing 100876, China; 2024140233@bupt.cn (Y.Q.); guoyijun@bupt.edu.cn (Y.G.); jjhao@bupt.edu.cn (J.H.); 2 Business School, Beijing Language and Culture University, Beijing 100083, China

**Keywords:** non-terrestrial network, edge computing, task offloading and resource allocation, deep reinforce learning

## Abstract

Leveraging non-terrestrial networks for edge computing is crucial for the development of 6G, the Internet of Things, and ubiquitous digitalization. In such scenarios, diverse tasks often exhibit continuously distributed attributes, while existing research predominantly relies on qualitative thresholds for task classification, failing to accommodate quantitatively continuous task requirements. To address this issue, this paper models a multi-task scenario with continuously distributed attributes and proposes a three-tier cloud-edge collaborative offloading architecture comprising UAV-based edge nodes, LEO satellites, and ground cloud data centers. We further formulate a system cost minimization problem that integrates UAV network load balancing and satellite energy efficiency. To solve this non-convex, multi-stage optimization problem, a two-layer multi-type-agent deep reinforcement learning (TMDRL) algorithm is developed. This algorithm categorizes agents according to their functional roles in the Markov decision process and jointly optimizes task offloading and resource allocation by integrating DQN and DDPG frameworks. Simulation results demonstrate that the proposed algorithm reduces system cost by 7.82% compared to existing baseline methods.

## 1. Introduction

With the advancement of communication and artificial intelligence technologies, the growing computational demands of intelligent tasks have surpassed the capabilities of traditional cloud computing, spurring increased attention to mobile edge computing (MEC) [1]. By deploying computational resources closer to user devices, MEC offers a denser distribution of computing nodes, better meeting requirements for high computational capacity and low-latency processing [2,3]. However, terrestrial networks face coverage limitations, especially in remote or inaccessible regions such as rural areas, deserts, and oceans. Integrating non-terrestrial components can enhance the performance of ground-based networks in terms of capacity, coverage, and latency, thereby extending the service boundaries of MEC [4].

Non-Terrestrial Networks (NTNs), incorporating satellites, HAPs, and UAVs, are emerging as key complements to terrestrial networks in 5G-Advanced and 6G evolution [5]. They provide essential edge computing with real-time capabilities in remote, disaster-affected, or maritime areas where ground infrastructure is unavailable. For instance, in disaster-stricken regions, UAVs can be rapidly deployed to form an aerial network, collecting sensor data and processing tasks locally, offloading to neighboring UAVs, or forwarding to LEO satellites based on computational and latency requirements. However, developing diverse NTN applications faces challenges such as limited computational and transmission resources of UAVs and difficulties in maintaining network stability and load balancing in multi-UAV collaboration [6]. Therefore, optimizing task offloading and resource allocation in NTN-enabled edge computing remains a critical and unresolved issue.

Task offloading in edge computing is generally approached through two paradigms: model-based online optimization and data-driven learning. The former employs methods such as dual-time-scale Lyapunov optimization [7], branch-and-price and greedy algorithms in fog systems [8], and multi-user scheduling mechanisms [9] to jointly optimize offloading and resource allocation under known system models. However, in highly dynamic non-terrestrial environments, accurate system modeling is often infeasible, motivating a shift toward deep reinforcement learning (DRL). Recent works such as [10,11] have applied DRL to integrated terrestrial–non-terrestrial settings, addressing problems such as joint optimization and distributed offloading. Notably, while [11] operates in discrete action spaces, our method supports continuous control for finer decision-making.

Existing studies commonly treat joint optimization of task offloading and resource allocation as key to enhancing system performance in NTN-based edge computing. Ref. [12] proposed a blockchain-integrated NTN framework with a customized consensus mechanism for joint offloading and resource allocation. Ref. [13] developed a priority-aware transmission scheme and a DDPG-based algorithm to maximize average system utility in scenarios involving tasks of varying priorities. Ref. [14] introduced a traffic-aware layered offloading framework for environments with significant load fluctuations, while [15] designed a scalable task scheduling solution adaptable to diverse NTN task scenarios.

However, real-world intelligent environments, such as smart agriculture [16], involve multiple tasks with overlapping attributes (e.g., computational load and latency sensitivity) distributed continuously, making fixed offloading strategies based solely on task type impractical. Moreover, competition for limited system resources further complicates multi-task offloading, an aspect not sufficiently addressed in the literature. Additionally, current studies lack integrated metrics that jointly evaluate the stability of aerial and space-based networks. UAV networks prioritize load balancing, whereas satellite networks—constrained by limited battery life and high replacement costs—require precise energy management [17]. Existing metrics often address these aspects in isolation, leading to inefficient resource utilization and compromised system-wide performance.

To address these challenges, this paper proposes a computation offloading and resource allocation strategy for continuous distributed tasks in a non-terrestrial network comprising multi-UAV and low earth orbit (LEO) satellite systems serving ground users. A collaborative three-tier cloud edge offloading architecture is designed for continuous task scenarios. Additionally, by accounting for UAV networking instability and the irreplaceable battery constraints of satellites, a composite metric evaluating UAV load balancing and LEO satellite energy consumption control is formulated, leading to a system cost minimization problem. To solve this problem, the two-layer based on the multi-type-agent deep reinforcement learning (TMDRL) algorithm is proposed. Simulation results demonstrate that the algorithm achieves optimal performance with minimal training overhead, exhibiting significant advantages over existing approaches.

The remainder of this paper is organized as follows: Section 2 introduces the system model. Section 3 formulates the optimization problem. Section 4 presents the proposed algorithm. Section 5 provides simulation results, and the paper is concluded in Section 6.

## 2. System Model

**Figure 1:**
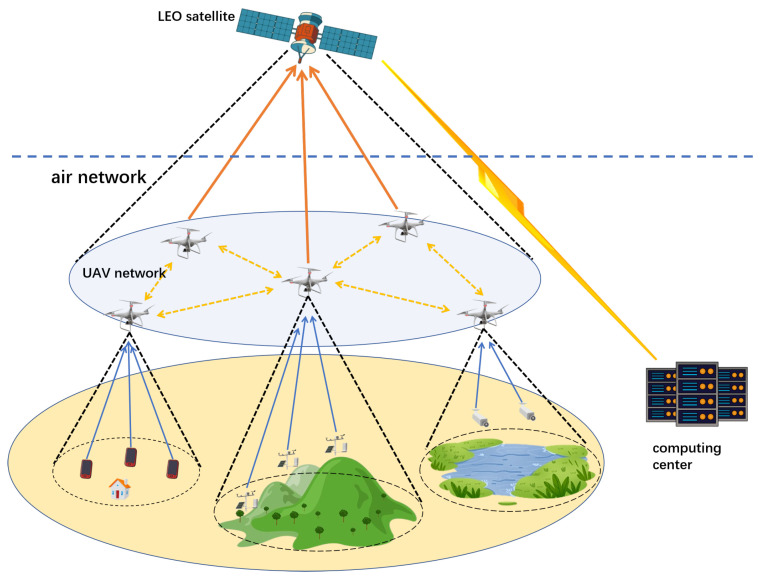
Scenario diagram of a non-terrestrial edge computing network.

This paper adopts a heterogeneous non-terrestrial network comprising *N* UAVs, one LEO satellite, and a remote ground computing center, where $V_{n}$ denotes the n-th UAV, and $E_{n,i}$ denotes the i-th equipment in region n. As shown in Figure 1, the ground is partitioned into *N* regions, each with *I* task-generating user equipment (UE)/IoT devices. The *N* pre-deployed UAVs form a fixed-topology network at low altitudes, while LEO satellites provide wide-area coverage with substantial computing resources, and UAVs enable low-latency edge computation via mobility. All aerial positions remain constant per time frame given brief task execution [18]. Task offloading follows: (1) Each device offloads to only one UAV; (2) a single UAV serves multiple devices via multi-access techniques in its coverage area.

At a given moment, ground devices simultaneously generate batches of tasks with varying attributes, requiring offloading to the space-air-ground integrated network for edge computation. This network employs a three-tier hierarchical processing architecture. The optimal offloading path for each task is dynamically determined based on its computational load, latency sensitivity, and real-time network conditions, leading to three possible processing outcomes: UAV Network Processing, which means tasks uploaded from ground equipment to their serving UAV may be computed locally by that UAV or forwarded to other neighboring UAVs. LEO Satellite Processing, which means tasks can be offloaded to the LEO satellite’s computational cache queue for processing when they require more substantial computational resources than the UAV network can provide. Ground computing center processing, which means tasks may be routed through the LEO satellite to the remote ground computing center for execution. This path is typically reserved for tasks that are highly computation-intensive but have relaxed latency constraints, making use of the center’s vast cloud computing capabilities.

### 2.1. Continuous Task Model

**Figure 2:**
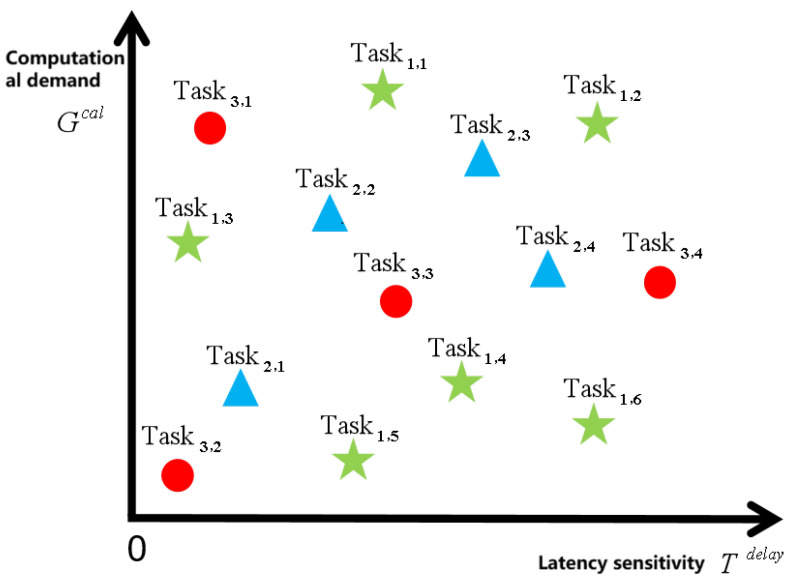
Schematic of the distribution of task attribute values over successive intervals.

This study characterizes a scenario where task attribute values follow a continuous distribution, thereby realistically reflecting the diverse requirements of tasks in complex environments. Each ground device ${\mathrm{Equip}}_{n,i}$ generates a task ${\mathrm{Task}}_{n,i}$, whose attributes are modeled as a two-tuple ${\mathrm{Task}}_{n,i}=\left(T_{n,i}^{\mathrm{delay}},G_{n,i}^{\mathrm{cal}}\right)$, representing latency sensitivity and computational workload, respectively. As illustrated in Figure 2, each task can be represented as a point in a 2D plane, where points of the same shape denote tasks generated by the same device cluster. The computational complexity and latency sensitivity of tasks in the model are distributed in continuous intervals, which can accurately characterize the processing requirements of diverse tasks in remote areas or outdoor environments. For example, tasks such as instant messaging and video stream analysis require extremely high real-time performance, and the latency usually needs to be controlled within 100 ms. However, for pre-processing and analysis of image or environmental information generated by ground equipment, the latency sensitivity can be relaxed to several hundred to several thousand ms according to specific needs [19]. This modeling method elevates the resource allocation problem from traditional finite combination optimization to infinite-dimensional decision space optimization, while significantly enhancing the model’s adaptability and generalization ability to different application scenarios.

### 2.2. Communication Model

This work focuses on the uplink offloading and computation phases. The network architecture comprises four distinct communication links: the ground-to-air (G2A) link between terrestrial devices and UAVs, the air-to-air (A2A) inter-UAV links, the air-to-space (A2S) links connecting UAVs to the LEO satellite, and the space-to-ground (S2G) link from the LEO satellite to the terrestrial computing center. The channel models for these links are specified in the following Table 1.

The proposed model accounts for the unique propagation characteristics of near-ground space environments, where terrain obstructions (mountains, vegetation, and human-made structures) necessitate modeling the ground-to-air (G2A) link as a probabilistic line-of-sight (LoS) channel, while all other links (A2A, A2S, and S2G) adopt conventional LoS channel models. Focusing on large-scale fading effects, the analysis primarily considers path loss-induced channel attenuation while neglecting small-scale fading, with additional shadowing effects incorporated for G2A links. The communication performance metrics—including transmission rate, latency, and energy consumption—are formally characterized as follows.

#### 2.2.1. Ground-to-Air Link

The distance between a ground device and a UAV for task transmission can be expressed as

(1) $$d_{n,i}^{G2A}=\sqrt{H_{0}^{2}+{∥s_{n,i}-u_{n}∥}^{2}}.$$

For a probabilistic line-of-sight (LoS) channel that considers shadowing effects, the communication power gain between the ground device and the UAV is given by

(2) $$h_{n,i}^{G2A}=L_{n,i}^{\mathrm{path}}+X_{\sigma },$$

where $X_{\sigma }$ represents the shadowing random variable following a normal distribution $X_{\sigma }∼N\left(0,\sigma^{2}\right)$, and $L_{n,i}^{\mathrm{path}}$ denotes the path loss composed of both line-of-sight (LoS) and non-line-of-sight (NLoS) components, expressed as [20]

(3) $$L_{n,i}^{\mathrm{path}}=P_{n,i}^{\mathrm{Los}}L_{n,i}^{\mathrm{Los}}+\left(1-P_{n,i}^{\mathrm{Los}}\right)L_{n,i}^{\mathrm{NLos}}.$$

The line-of-sight path loss is expressed as

(4) $$L_{n,i}^{\mathrm{Los}}=\frac{\beta_{0}}{H_{0}^{2}+{∥s_{n,i}-u_{n}∥}^{2}},$$

where $\beta_{0}$ denotes the channel power gain at the reference distance of 1 m. According to Shannon’s capacity formula, the transmission rate from the ground device to the UAV for task offloading is given by

(5) $$R_{n,i}^{G2A}=B^{G2A}log\left(1+\frac{p^{G2A}h_{n,i}^{G2A}}{\sigma^{2}}\right).$$

#### 2.2.2. Air-to-Air Link

All communication links among UAVs form a network graph, in which the communication forwarding route between any two nodes is determined using the shortest path algorithm, namely Dijkstra’s algorithm. Then, the distance of an edge in the graph can be expressed as

(6) $$d_{a_{s},a_{s+1}}^{A2A}=∥u_{a_{i}}-u_{a_{i+1}}∥.$$

Since air-to-air (A2A) links are line-of-sight (LoS) channels, the transmission rate over a given edge is given by

(7) $$R_{n,i,a_{s}}^{A2A}=B^{A2A}log\left(1+\frac{p^{A2A}h_{a_{s},a_{s+1}}^{A2A}}{\sigma^{2}}\right).$$

#### 2.2.3. Air-to-Space Link

Similarly, the air-to-space (A2S) link is also a line-of-sight (LoS) channel. The task transmission rate from the UAV to the LEO satellite is given by [21]

(8) $$R_{n,i}^{A2S}=B^{A2S}log\left(1+\frac{p^{A2S}h_{n}^{A2S}}{\sigma^{2}}\right).$$

#### 2.2.4. Space-to-Ground Link

**Figure 3:**
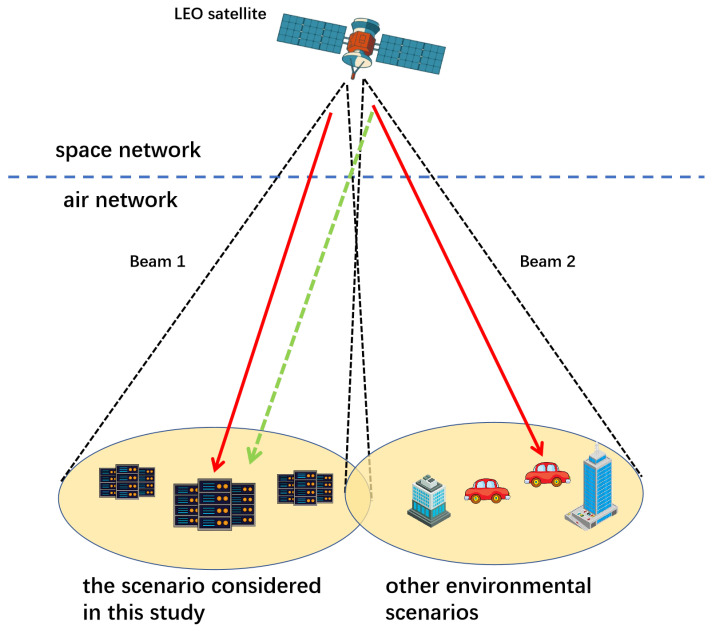
Illustration of multi-beam interference in space-to-ground links.

To address the co-channel interference in the space-to-ground link (as indicated by the dashed lines in Figure 3), the satellite downlink offloading in our study scenario is subject to co-channel interference from adjacent beams serving other scenarios. To quantify this interference, we construct an interference coefficient matrix G based on the normalized gain of the satellite transmitting antenna,

(9) $$G=\frac{1}{G_{\max}}\left[\begin{array}{cccc} G_{1,1} & G_{1,2} & \ldots & G_{1,k}\\ G_{2,1} & G_{2,2} & \ldots & G_{2,k}\\ \vdots & \vdots & \ddots & \vdots \\ G_{k,1} & G_{k,2} & \ldots & G_{k,k} \end{array}\right],$$

where *K* denotes the number of satellite beams, $G_{\max}$ represents the maximum transmit antenna gain of the satellite, and $G_{a,1}$ indicates the interference from beam *a* in the direction of beam 1 within the target scenario.

The useful signal gain received by the computing center is given by

(10) $$h_{n,i}^{S2G\text{-}U}=\frac{\beta_{0}}{H_{1}^{2}+{∥1-c∥}^{2}}+G_{\max}.$$

The interference signal gain from other beams received by the computing center is given by

(11) $$I_{n,i}^{S2G\text{-}I}=\sum_{a=1}^{K}p_{a}h_{n,i,a}^{S2G\text{-}I}.$$

The transmission power from the LEO satellite to the computing center is given by

(12) $$R_{n,i}^{S2G}=B^{S2G}\log\left(1+\frac{p_{n,i}^{S2G}h_{n}^{A2S}}{\sigma^{2}+I_{n,i}^{S2G\text{-}I}}\right).$$

### 2.3. Computational Model

This chapter investigates a three-tier computing architecture comprising unmanned aerial vehicles (UAVs), LEO satellites, and ground computing centers. The resource constraints of each computing entity are modeled as follows.

#### 2.3.1. UAV Computing Node

Commercial survey drones equipped with low-power embedded GPU/FPGA units, delivering typical computational capacity of 10 GFLOPS. Each UAV’s onboard computing resources have a maximum processing rate of $R_{\max}^{\mathrm{UAV}}$. The computation latency for tasks is given by $t_{n,i}^{\mathrm{cal}}=G_{n,i}^{\mathrm{cal}}/R_{n,i}^{\mathrm{UAV}}$, where parallel task execution is supported through allocated computing resource partitioning.

#### 2.3.2. Ground Computing Center

Assumed to possess sufficient computational resources (effectively infinite capacity), where processing latency is dominated by transmission delays with negligible local computation time.

#### 2.3.3. LEO Satellite Node

Onboard servers with computational capacity reaching hundreds of GFLOPS, suitable for medium-to-high load tasks like video analytics. Due to energy constraints and multi-user sharing, satellites adopt sequential task scheduling, where tasks queue for execution. The maximum computation rate is denoted as $R_{\max}^{\mathrm{LEO}}$, with single-task processing at any given time.

The computational power consumption for LEO satellite task processing is expressed as [22]

(13) $$p_{n,i}^{\mathrm{LEO}}=K^{\mathrm{LEO}}{\left(\frac{R_{n,i}^{\mathrm{LEO}}}{\mathrm{IPC}\times N^{\mathrm{LEO}}\times v}\right)}^{3},$$

where $K^{\mathrm{LEO}}$ is a scaling factor related to the chip hardware, $IPC,N^{\mathrm{LEO}},v$ denotes the number of instructions per clock cycle, the number of processor cores, and the number of floating-point operations per instruction, respectively, which are also constants related to the hardware. latency of processing tasks for orbiting satellites $t_{n,i}^{\mathrm{cal}}=G_{n,i}^{\mathrm{cal}}/R_{n,i}^{\mathrm{LEO}}$, computational energy consumption $q_{n,i}^{\mathrm{cal}}=p_{n,i}^{\mathrm{LEO}}t_{n,i}^{\mathrm{cal}}$.

### 2.4. Load Balancing and Energy Control Model

All tasks in this chapter are generated simultaneously by the ground equipment, and a timeline is created on this origin. Assume that the moment when a task starts to be transmitted from the ground equipment is noted as $t_{0}$, the first task is counted by the UAV as $t_{1}$, and the moment when the UAV finishes processing the last task is $t_{2}$. Then at a certain moment *t*, the ${\mathrm{UAV}}_{n}$ processes *w* tasks at the same time, $G_{j}^{\mathrm{cal}}$ is the processing rate allocated by the UAV for one of the tasks, and then the computation-resource utilization ratio (CUR) at the moment *t* is [23]

(14) $$U_{n}^{C}\left(t\right)=\sum_{j=1}^{w}\frac{G_{j}^{\mathrm{cal}}}{R^{\mathrm{UAV}}}.$$

To accurately characterize the overall load of a UAV communication network, it is insufficient to focus solely on the resource utilization of individual nodes; the resource utilization of neighboring UAVs in communication must also be taken into account. The local computation-resource utilization ratio (LCUR) of a central node is defined as the weighted sum of the utilization rates of both the central node and its adjacent nodes, which can be expressed as

(15) $$U_{n}^{\mathrm{LC}}\left(t\right)=\alpha U_{n}^{C}\left(t\right)+\left(1-\alpha \right)\sum_{n=1}^{m}U_{n,m}^{C}\left(t\right).$$

By adjusting α, the value of ${\mathrm{LCUR}}_{n}\left(t\right)$ can be modified to reflect different weightings for either the central node or the local region’s load intensity, thereby accommodating networks with varying parameters and scales. The average computation-resource utilization ratio across the entire network, denoted as the average computation-resource utilization ratio (ACUR), is given by

(16) $$U^{\mathrm{AC}}\left(t\right)=\frac{1}{N}\sum_{n=1}^{N}U_{n}^{C}\left(t\right).$$

Finally, we define the UAV network load balancing indicator (ULBI) as one of the key components of the total system consumption. For task *i* in region *n*, the load balancing indicator for task offloading and resource allocation decision is defined as

(17) $$I_{n,i}^{\mathrm{ULB}}=\frac{1}{t_{n,i}^{\mathrm{end}}-t_{n,i}^{\mathrm{begin}}}\int_{t_{n,i}^{\mathrm{begin}}}^{t_{n,i}^{\mathrm{end}}}\frac{\sum_{n=1}^{N}{\left|U_{n}^{\mathrm{LC}}\left(t\right)-U^{\mathrm{AC}}\left(t\right)\right|}^{2}}{N}dt,$$

where $t_{n,i}^{\mathrm{begin}}$ and $t_{n,i}^{\mathrm{end}}$ are the start time and completion time of task n,i, respectively. And the ULBI of the whole network is calculated by

(18) $$I^{\mathrm{ULB}}=\sum_{n=1}^{N}\sum_{i=1}^{I}I_{n,i}^{\mathrm{ULB}}.$$

**Figure 4:**
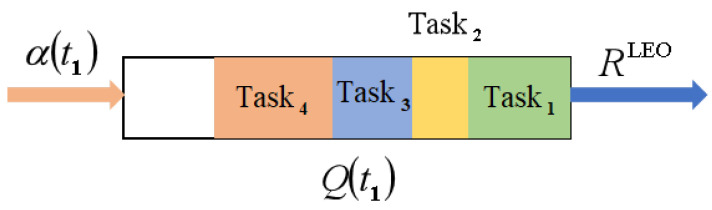
Satellite computation cache queue model.

Due to the need to control the energy consumption of the satellite while processing a large number of tasks, there exist two cache queue models on the satellite: a computation queue and a transmission queue [14]. As an example, the computation cache queue is illustrated, as shown in Figure 4.

The update formula for the queue data amount is given by

(19) $$Q\left(t_{2}\right)=\max\left\{Q\left(t\right)-R^{\mathrm{LEO}}\left(t_{2}-t_{1}\right)+a\left(t_{0}\right),0\right\}.$$

Similarly to the task execution buffer queue on satellites, the battery energy of LEO satellites can also be modeled as an energy pool consisting of a consumption side and a charging side. The consumption side includes computational and transmission energy expenditures, while the charging side comprises the solar panel power generation system onboard the satellite. The update formula for the battery energy in LEO satellites is given by

(20) $$C\left(t_{2}\right)=C\left(t_{1}\right)+\int_{t_{1}}^{t_{2}}\eta_{s}\gamma Mdt-\int_{t_{1}}^{t_{2}}p^{\mathrm{LEO}}+p^{S2G}dt,$$

where $\eta_{s}$ represents the photovoltaic conversion efficiency of the solar panels, γ denotes the solar irradiance intensity, and *M* is the area of the solar panel array.

For lithium-ion batteries powering LEO satellites, their performance and operational lifespan largely determine the satellite’s overall functionality and service duration. However, the cycle life of these batteries is not fixed but rather constrained by multiple interdependent factors. The most significant influencing factor is the depth of discharge (DOD), which represents the proportion of discharged capacity relative to the battery’s rated capacity,

(21) $$D\left(t\right)=\frac{C_{\max}-C\left(t\right)}{C_{\max}},$$

where $C_{\max}$ denotes the battery’s maximum capacity and C(t) represents the remaining energy at time *t*. According to existing research [24], the battery lifetime degradation function can be expressed as

(22) $$f\left(D\left(t\right)\right)=10^{A(D(t)-1)}\left(1+A\ln10\cdot D(t)\right).$$

By performing a weighted summation of task execution energy consumption and battery lifetime degradation, we establish an evaluation metric for LEO satellite energy management, termed the LEO energy control indicator (LECI). The LECI for task *i* in region *n* is defined as

(23) $$I_{n,i}^{\mathrm{LEC}}=\beta \int_{t_{n,i}^{\mathrm{begin}}}^{t_{n,i}^{\mathrm{end}}}\left(p^{\mathrm{LEO}}+p^{S2G}\right)dt+\left(1-\beta \right)\int_{t_{n,i}^{\mathrm{begin}}}^{t_{n,i}^{\mathrm{end}}}f\left(D\left(t\right)\right)dt.$$

And the LECI of the whole network is calculated by

(24) $$I^{\mathrm{LEC}}=\sum_{n=1}^{N}\sum_{i=1}^{I}I_{n,i}^{\mathrm{LEC}}.$$

## 3. Task Optimization Model for Non-Terrestrial Networks with Resource Coordination

Based on the aforementioned system model, this chapter formulates an optimization problem aimed at minimizing the energy consumption of the non-terrestrial network. By jointly optimizing offloading decisions and resource allocation, the system performance is enhanced. In the current simulation experiments, the system is required to provide edge computing for all tasks, with the optimization objective being the minimization of total system consumption after completing all tasks. This objective is defined as the weighted sum of the UAV network load balancing indicator and the LEO satellite energy control indicator, which can be expressed as

(25) $$U=\Phi_{1}\cdot I^{\mathrm{ULB}}+\Phi_{2}\cdot I^{\mathrm{LEC}}.$$

Under these conditions, the objective of optimization of this chapter is to minimize task processing consumption in the non-terrestrial network by jointly optimizing the task offloading decision variable $X_{n,i,k}$, the computing resources allocated by UAV $R_{n,i}^{\mathrm{UAV}}$, the computing resources allocated by LEO satellite $R_{n,i}^{\mathrm{LEO}}$ and the transmission power allocated by LEO satellite $P_{n,i}^{S2G}$. Here, $X_{n,i,k}$ indicates whether the *i*-th task in region *n* is offloaded to computing node *k*, where $k\in K=\left\{1,2,\ldots ,N,N+1,N+2\right\}$ with the first *N* nodes representing UAVs, node N+1 representing the LEO satellite, and node N+2 representing the remote computing center. The optimization problem is formulated as follows.

$$\begin{array}{ccc} & \left(P1\right):\min_{X,R^{\mathrm{UAV}},R^{\mathrm{LEO}},P}U & \end{array}$$

(26a) $$\begin{array}{cccc} s.t.\; & x_{n,i,k}\in \left\{0,1\right\}, & & \forall n,i,k \end{array}$$

(26b) $$\begin{array}{cccc} & \sum_{k=1}^{N+2}x_{n,i,k}\leq 1, & & \forall n,i \end{array}$$

(26c) $$\begin{array}{cccc} & \sum_{n=1}^{N}\sum_{i=1}^{I}R_{n,i}^{\mathrm{UAV}}x_{n,i,k}\leq R_{\max}^{\mathrm{UAV}}, & & \forall k \end{array}$$

(26d) $$\begin{array}{cccc} & R_{n,i}^{\mathrm{LEO}}\leq R_{\max}^{\mathrm{LEO}}, & & \forall n,i \end{array}$$

(26e) $$\begin{array}{cccc} & P_{n,i}^{S2G}\leq P_{\max}^{\mathrm{LEO}}, & & \forall n,i \end{array}$$

(26f) $$\begin{array}{ccc} & Q\left(t\right)\leq Q_{\max}, & \end{array}$$

(26g) $$\begin{array}{cccc} & t_{n,i}^{G2A}+t_{n,i}^{A2A}+t_{n,i}^{\mathrm{cal}}\leq T_{n,i}^{\mathrm{delay}}, & & \forall n,i \end{array}$$

(26h) $$\begin{array}{cccc} & t_{n,i}^{G2A}+t_{n,i}^{A2S}+t_{n,i}^{\mathrm{wait}}+t_{n,i}^{\mathrm{cal}}\leq T_{n,i}^{\mathrm{delay}}, & & \forall n,i \end{array}$$

(26i) $$\begin{array}{cccc} & t_{n,i}^{G2A}+t_{n,i}^{A2S}+t_{n,i}^{\mathrm{wait}}+t_{n,i}^{S2G}\leq T_{n,i}^{\mathrm{delay}}, & & \forall n,i \end{array}$$

The optimization problem incorporates nine key constraints to ensure system feasibility and performance. Constraints (26a) and (26b) govern task offloading, where (26a) enforces binary offloading decisions and (26b) guarantees each task is exclusively assigned to one computing node. Resource capacity limitations are addressed through constraint (26c), which prevents UAVs from exceeding their maximum computational capacity when allocating resources to multiple tasks, while constraints (26d) and (26e) similarly restrict the LEO satellite’s computational and transmission resources to their respective maximum capacities. The system’s data management is regulated by constraint (26f), which maintains the satellite’s queue buffer within permissible limits. Constraints (26g)–(26i) are task execution delay constraints, corresponding to three possible offloading paths: drone network, low orbit satellite, and ground computing center, ensuring that the total task execution delay does not exceed the delay sensitivity threshold of the task. These constraints work in concert to maintain system stability while achieving the optimization objectives.

## 4. Algorithmic Solution

### 4.1. Deep Reinforcement Learning Based on Multi-Agent

To address this optimization problem, this paper formulates the task offloading and resource allocation process in non-terrestrial network edge computing as a multi-agent collaborative decision-making framework, where distinct intelligent agents are designed according to their respective optimization variables. The proposed system deploys four types of decision-making agents across UAVs and LEO satellites. Specifically, each UAV is equipped with a Task Offloading agent (TO agent) that determines optimal computing nodes for task processing and a UAV Resource Allocation agent (URA agent) that manages computing resource distribution for UAV-hosted tasks. Similarly, the LEO satellite incorporates two specialized agents: an LEO Computing Resource Allocation agent (LCRA agent) that handles computation resource allocation for satellite-processed tasks and an LEO Transmission Resource Allocation agent (LTRA agent) responsible for optimizing transmission resource allocation when tasks are offloaded to ground computing centers.

This study models the task offloading and resource allocation process for edge computing in NTN as a Partially Observable Markov Decision Process (POMDP), represented by the following sextuple:

(27) P=(S,A,T,R,O,γ).

The global state space, denoted by *S*, represents the complete set of state information for both the UAV network and the LEO satellite within the integrated space-air network at a given timestep.

(28) $$S=\left\{s_{1},s_{2},s_{3},\ldots ,s_{N},s_{\mathrm{cal}}^{\mathrm{LEO}},s_{\mathrm{trans}}^{\mathrm{LEO}},s_{\mathrm{life}}^{\mathrm{LEO}},s^{\mathrm{Task}}\right\}.$$

The action space *A* is defined as the Cartesian product of the action spaces of all four agent types in the system.

(29) $$A=\left\{A^{\mathrm{TO}},A^{\mathrm{URA}},A^{\mathrm{LCRA}},A^{\mathrm{LTRA}}\right\}.$$

The state transition function *T* defines the probability of transitioning from one state to another when taking a given action in a specific state. In this study, since state transitions are uniquely determined when both the current state and action are specified, all transition probabilities are equal to 1. The reward function *R* represents the immediate feedback or reward that an agent receives from the environment after executing an action.

*O* represents the partial observation space, with its four elements, respectively, denoting the environmental information observable by the four types of intelligent agents.

(30) $$O=\left\{O^{\mathrm{TO}},O^{\mathrm{URA}},O^{\mathrm{LCRA}},O^{\mathrm{LTRA}}\right\}.$$

Next, the design of the partial observation spaces and action spaces for each intelligent agent is detailed as follows:

The partial observation space for the task offloading agent (TO agent) of the unmanned aerial vehicle is denoted as $O^{\mathrm{TO}}=\left\{s_{1},s_{2},s_{3},\ldots s_{N},s_{\mathrm{cal}}^{\mathrm{LEO}},s_{\mathrm{trans}}^{\mathrm{LEO}},s^{\mathrm{Task}}\right\}$. This agent can observe the remaining computational resources of all UAVs, the data volume in the computation and transmission queues of the low-earth orbit satellite, as well as the attribute information of the tasks being processed. The partial observation space for the UAV resource allocation agent (URA agent) is denoted as $O^{\mathrm{URA}}=\left\{s_{1},s_{2},s_{3},\ldots ,s_{N},s^{\mathrm{Task}}\right\}$. Based on the remaining computational resources of all UAVs and the attributes of the tasks, this agent allocates the computational power for task processing at its own node.

The partial observation space for the low-earth orbit satellite computational resource allocation agent (LCRA agent) is denoted as $O^{\mathrm{LCRA}}=\left\{s_{\mathrm{cal}}^{\mathrm{LEO}},s_{\mathrm{life}}^{\mathrm{LEO}},s^{\mathrm{Task}}\right\}$. This agent makes decisions on computational resource allocation based on the data volume in the satellite’s computation queue, the lifetime degradation status, and the attributes of the tasks.

The partial observation space for the low-earth orbit satellite transmission resource allocation agent (LTRA agent) is denoted as $O^{\mathrm{LTRA}}=\left\{s_{\mathrm{trans}}^{\mathrm{LEO}},s_{\mathrm{life}}^{\mathrm{LEO}},s^{\mathrm{Task}}\right\}$. This agent makes decisions on transmission resource allocation based on the data volume in the satellite’s transmission queue, the lifetime degradation status, and the attributes of the tasks.

The action space of the TO agent is $A^{\mathrm{TO}}=\left\{1,2,3,\ldots ,N,N+1,N+2\right\}$, and the first *N* digits indicate that the task is unloaded to the UAV with the corresponding number for execution, N+1 indicates that the task is unloaded to the LEO satellite for execution, and N+2 indicates that the task is unloaded to the ground computing center for execution. The action space of the URA agent is $A^{\mathrm{URA}}=\left\{R^{\mathrm{UAV}}\right\}$. $R^{\mathrm{UAV}}$ is the computational rate allocated by the UAV for the task. The action space of the LCRA agent is $A^{\mathrm{LCRA}}=\left\{R^{\mathrm{LEO}}\right\}$. $R^{\mathrm{LEO}}$ is the computational rate allocated by the LEO satellite for the task. The action space of the LTRA agent is $A^{\mathrm{LTRA}}=\left\{p^{S2G}\right\}$. $p^{S2G}$ is the transmission power allocated by the LEO satellite for the task.

### 4.2. Design of the Reward Function

The design of the reward function consists of two parts: the first is the positive reward for the optimization objectives, which reflects the contribution of an action, such as executing a task, towards the primary optimization goal—minimizing task processing consumption. The second part is the negative penalty for constraint violations, where an action that exceeds the constraint limits receives significant negative feedback from the environment.

The optimization objective of this study is to minimize the load balancing index and energy consumption control index. However, in reinforcement learning, the goal is to maximize the reward value. Therefore, exponential transformation is applied to these indices to form a positive incentive, which is defined as

(31) $$M_{n,i}^{\mathrm{motivate}}=\omega \cdot e^{\mu (\Phi_{1}I_{n,i}^{\mathrm{ULB}}+\Phi_{2}I_{n,i}^{\mathrm{LEC}})}-b.$$

The design of negative rewards focuses on the constraint conditions. Among the nine constraint conditions of the optimization problem, constraints (26a)–(26c) and (26e) are fixed by the range of values in the action space, so no violations will occur. The remaining constraint conditions require the design of specific penalty functions. In this study, the penalty value is set to be proportional to the extent by which the threshold is exceeded. The negative penalty is defined as

(32) $$\begin{array}{cc} g_{1} & =\left(\sum_{k=1}^{N}R_{n,i}^{\mathrm{UAV}}x_{n,i,k}\right)-R_{\max}^{\mathrm{UAV}}, \end{array}$$

(33) $$\begin{array}{cc} g_{2} & =Q\left(t\right)-Q_{\max}, \end{array}$$

(34) $$\begin{array}{cc} g_{3} & =t_{n,i}^{\mathrm{total}}-T_{n,i}^{\mathrm{delay}}, \end{array}$$

(35) $$\begin{array}{cc} P_{n,i}^{\mathrm{penalty}} & =\phi_{1}\max\left(g_{1},0\right)+\phi_{2}\max\left(g_{2},0\right)+\phi_{3}\max\left(g_{3},0\right). \end{array}$$

The reward function is the difference between the positive incentive and the negative penalty, which is defined as

(36) $$r_{n,i}=M_{n,i}^{\mathrm{motivate}}-P_{n,i}^{\mathrm{penalty}}.$$

### 4.3. Two-Layer Based on Multi-Type-Agent Deep Reinforcement Learning Algorithm

**Figure 5:**
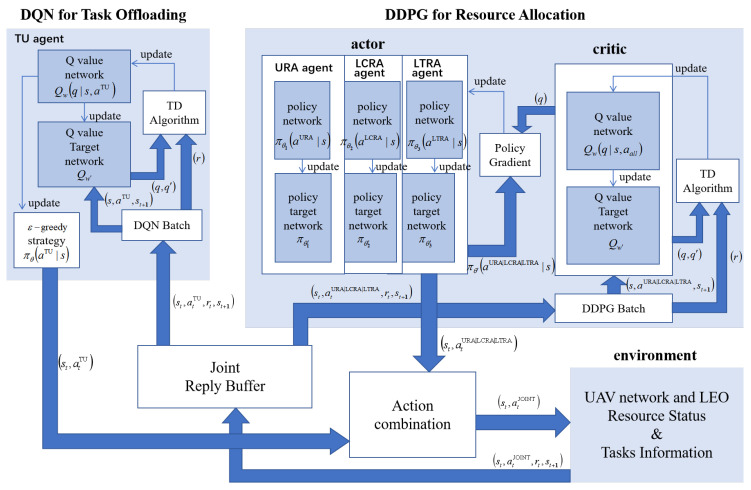
Framework of the TMDRL algorithm.

Based on the multi-agent framework and key elements of Markov decision processes, this study proposes a two-layer multi-type-agent deep reinforcement learning (TMDRL) algorithm for task offloading and resource allocation. The TMDRL architecture integrates four core components: a DQN network for discrete action selection, a DDPG network for continuous control, a shared experience replay buffer that couples both networks, and an action merging module that synthesizes the final decisions. The complete network structure is illustrated in Figure 5.

#### 4.3.1. DQN Network

The DQN architecture comprises two structurally identical neural networks: an online Q-network and a target Q-network. The online Q-network takes state-action pairs $\left(s_{t},a_{t}\right)$ as input and outputs the corresponding Q-value $Q(s_{t},a_{t})$. The network parameters are updated through temporal difference (TD) learning, where a batch of transitions is sampled from the experience replay buffer. The mean square error between the Q values predicted by the online network and the outputs of the target network for subsequent states serves as the loss function $L_{w}$, which is minimized by backpropagation to update the online Q network. The loss function can be described as

(37) $$\begin{array}{c} L_{w}=\frac{1}{N}\sum_{t=1}^{N}{\left(Q_{w}\left(s_{t},a_{t}\right)-\gamma \cdot Q_{w^{\prime }}\left(s_{t+1},a_{t+1}\right)+r_{t}\right)}^{2}. \end{array}$$

The task offloading action is determined by the classical greedy strategy. The action selection mechanism can be described as

(38) $$\begin{array}{c} a_{t}=\left\{\begin{array}{cc} \arg\max_{a_{t}}Q\left(s_{t},a_{t}\right), & w.p.\;1-\varepsilon ,\\ \mathrm{random}\;\mathrm{action}, & w.p.\;\varepsilon . \end{array}\right. \end{array}$$

#### 4.3.2. DDPG Network

DDPG adopts a distributed architecture with three independent policy networks and a centralized critic network. The three policy networks provide decision-making for the three agents: URA agent, LCRA agent, and LTRA agent. Each policy network consists of two components: a policy network and a target policy network. For the URA agent, its update iteration formula in the DDPG framework can be described as

(39) $$\theta_{t+1}^{\mathrm{URA}}=\theta_{t}^{\mathrm{URA}}+\tau \cdot ▽J\left(\theta \right).$$

The optimization objective is to maximize the state-action value output by the critic network. This can be represented as

(40) $$J\left(\theta \right)=E_{s∼D}\left[R(s,a)\right]=Q_{w}\left(s,a\right).$$

The policy gradient can be expressed as

(41) $$\begin{array}{cc} ▽J(\theta ) & =▽Q_{w}\left(s,a\right)=▽Q_{w}\left(s,\pi \left(s;\theta \right)\right)\\ \; & =\frac{\partial \pi (s;\theta )}{\partial \theta }\cdot \frac{\partial Q_{w}\left(s,a\right)}{\partial a}. \end{array}$$

The loss function can be defined as

(42) $$L_{\theta }=-Q_{\theta }\left(s,\pi \left(s,\theta \right)\right).$$

In DDPG, the Q-network update also uses the temporal difference (TD) method. However, in the loss function, the output of the target policy network is used as the input action for the target Q-network. This can be expressed as

(43) $$L_{w}=\mathrm{MSE}\left(Q_{w}\left(s_{t},a_{t}\right),Q_{w^{\prime }}\left(s_{t+1},\pi_{\theta^{\prime }}\left(s\right)\right)+r_{t}\right).$$

#### 4.3.3. Training Method and Execution Flow

In conventional reinforcement learning training, synchronous methods are typically employed. However, in the context of this study, agent-environment interactions must complete all tasks in a single uninterrupted sequence rather than interacting independently per task. Given the system architecture comprising two core networks (DQN and DDPG), four actor types, and two critics, we adopt an asynchronous training paradigm to balance task execution requirements with training efficiency. This asynchronicity manifests in three key aspects. The detailed procedure is presented in Algorithm 1.

First, the sequential coupling between agent-environment interactions and network parameter updates is decoupled, allowing task execution and network updates to proceed in parallel. Second, the inherent binding between action execution and immediate reward acquisition is separated. Since the load-balancing reward for any given task depends on the execution status of other concurrent tasks, our framework first processes all task-environment interactions to obtain new states, then conducts a unified secondary interaction phase to compute rewards. Third, the temporal dependency between DQN and DDPG updates is eliminated—both networks commence independent parameter training immediately upon experience collection, with only the DDPG internal actor-critic updates maintaining a fixed frequency synchronization.
**Algorithm 1** Two-layer Multi-agent Deep Reinforcement Learning for Task Offloading and Resource Allocation (TMDRL).**Require:** Initialize DQN and DDPG networks parameters: θ, $\theta^{\prime }$, *w*, $w^{\prime }$; Initialize joint experience replay buffer; Set target network update interval *step*; Set policy network update frequency *frequency* in DDPG; Initialize all physical parameters of the space-air network.**Ensure:** Optimized policy for task offloading and resource allocation.  1: **for** m=1 to *M* **do**            ▹ Training episode loop  2:       Generate N·I tasks, initialize $G^{\mathrm{cal}}$, $T^{\mathrm{delay}}$  3:       Initialize state space *S*, partial observation spaces *O*  4:       Initialize exploration factor ϵ, action noise ν  5:       **for** each task ${\mathrm{Task}}_{n,i}$ in order of arrival **do**  6:             TO agent on UAV selects offloading action $a_{i}^{\mathrm{TO}}$  7:             **if** $a_{i}^{\mathrm{TO}}\in \left\{1,\ldots ,N\right\}$ **then**                  ▹ Offload to UAV  8:                  URA agent selects computing resource action $a_{i}^{\mathrm{URA}}$  9:             **else if** $a_{i}^{\mathrm{TO}}=N+1$ **then**                ▹ Offload to LEO for computation 10:                  LCRA agent on LEO selects action $a_{i}^{\mathrm{LCRA}}$ 11:             **else if** $a_{i}^{\mathrm{TO}}=N+2$ **then**                ▹ Offload to GCC via LEO 12:                  LTRA agent on LEO selects action $a_{i}^{\mathrm{LTRA}}$ 13:             **end if** 14:             Merge actions: $a_{i}^{\mathrm{JOINT}}=a_{i}^{\mathrm{TO}}\cup a_{i}^{\mathrm{resource}}$ 15:             Execute $a_{i}^{\mathrm{JOINT}}$, observe next state $s_{t+1}$ 16:       **end for** 17:       Obtain rewards $r_{i}$ for all state-action pairs after all tasks are executed 18:       Store experiences $\left(s_{t},s_{t+1},a_{i}^{\mathrm{JOINT}},r_{i}\right)$ into joint replay buffer 19:       Sample a batch of *L* experiences from the replay buffer 20:       **for** *round* =1 to *L* **do**              ▹ DQN update thread 21:             Compute loss for DQN Q-network via TD error 22:             Update DQN Q-network parameters 23:             **if** *round* mod *step* =0 **then** 24:                  Update target Q-network: $Q_{w^{\prime }}\leftarrow Q_{w}$ 25:             **end if** 26:       **end for** 27:       **for** *round* =1 to *L* **do**                ▹ DDPG update thread 28:             Compute loss for DDPG critic network via TD error 29:             Update DDPG critic network parameters 30:             **if** *round* mod*step*=0 **then** 31:                  Update target critic network: $Q_{w^{\prime }}\leftarrow Q_{w}$ 32:             **end if** 33:             **if** *round* mod*frequency*=0 **then** 34:                  Compute policy gradient for actor network 35:                  Update DDPG actor network parameters 36:                  **if** *round* mod (*frequency*·*step*) =0 **then** 37:                       Update target actor network: $\pi_{\theta^{\prime }}\leftarrow \pi_{\theta }$ 38:                  **end if** 39:             **end if** 40:       **end for** 41: **end for**

## 5. Simulation Results and Analysis

### 5.1. Parameter Settings and Benchmark Algorithms

This section analyzes the performance of the proposed algorithm through numerical simulations. The simulation parameters and their corresponding values are summarized in Table 2. In addition to the proposed TMDRL algorithm, some benchmark algorithms are designed in this section.

#### 5.1.1. Algorithms for Task Offloading and Resource Allocation Using Deterministic Methods

In order to control the optimization variables, only one of task offloading or resource allocation is chosen for comparison in the comparison algorithms. The first one is a resource allocation algorithm using a task offloading strategy with proximity assignment + DDPG (DDPG-PR), where the tasks are processed by the UAVs in the area served and offloaded to satellites or computational centers only when the task data volume is above a certain threshold. The second comparison algorithm is the task offloading + greedy resource allocation strategy (DQN-GD) using DQN, where the greedy strategy refers to allocating the maximum remaining arithmetic or transmission resources for the current task.

#### 5.1.2. Algorithms with a Single-Layer Architecture and Fewer Types of Agents

The purpose is to demonstrate that in the staged task processing flow and different action spaces described in this article, the reinforcement learning architecture using a dual layer multi-agent is more effective. The first comparison algorithm is to remove the DQN for optimizing task offloading and to centralize the optimization of task offloading and resource allocation (DDPG) using only DDPG, in which the decisions of task offloading and resource allocation are made simultaneously, and then discretized fetching is performed for the output continuous task offloading actions. The second comparison algorithm is to keep the two-tier architecture and set only one intelligence RA agent in the DDPG to unify the resource allocation decisions on UAVs and satellites (TTDRL), which is compared with this algorithm to show the importance of setting up multiple types of intelligences for different action spaces.

### 5.2. Feasibility Analysis

**Figure 6:**
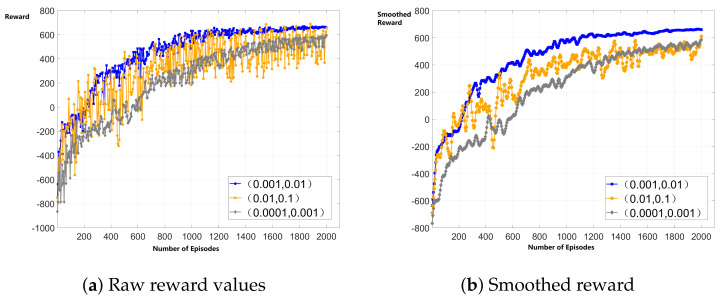
Convergence curves under different learning rates.

To validate algorithm convergence, we first tested the reward value progression of TMDRL under different learning rate combinations. As shown in Figure 6, with an actor-critic learning rate pair of (0.001, 0.01), the reward value rises rapidly within the first 1000 episodes, then stabilizes with reduced fluctuations, eventually converging around 650. Higher learning rates led to persistent oscillations, while lower rates resulted in undesirably slow convergence.

Furthermore, we compared the convergence episodes of TMDRL with two baseline reinforcement learning algorithms—DQN and DDPG. Simulation results show that DDPG converged the fastest, stabilizing after approximately 450 episodes, while DQN required around 520 episodes. In comparison, TMDRL converged at about 1500 episodes. This slower convergence can be attributed to its dual-loop decision-making architecture, which demands more training rounds to coordinate policy updates across different levels.

### 5.3. System Consumption

**Figure 7:**
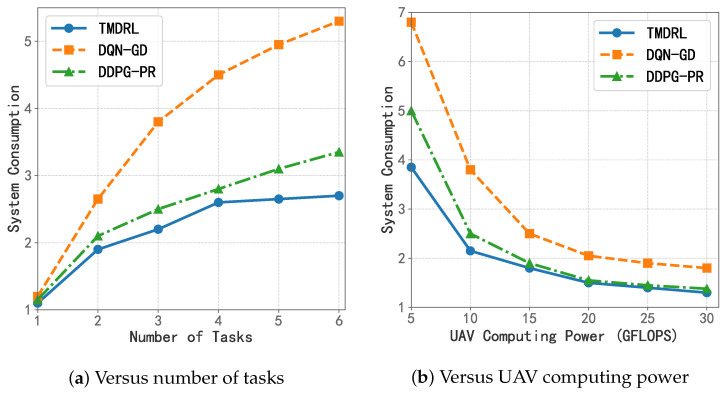
System consumption compared with the first group of algorithms.

As shown in Figure 7, the comparison with the two benchmark algorithms in the first group can clearly conclude the algorithmic advantage of TMDRL. For the DDPG-PR algorithm, which performs computational offloading with a fixed rule, and the DQN-GD algorithm, which performs resource allocation with a greedy strategy, TMDRL achieves significant advantages under different numbers of tasks and UAV arithmetic conditions, and the advantages are most prominent when the task data volume is large and the UAV arithmetic resources are small. When *I* = 3 and $R_{\max}^{\mathrm{UAV}}$ = 10 GFLOPS, TMDRL has 10.04% and 40.27% lower system consumption than DDPG-PR and DQN-GD, respectively, which indicates that the use of reinforcement learning intelligences iteratively for decision-making of multivariate variables, especially for continuous variables such as resource allocation, has a very good performance optimization performance.

**Figure 8:**
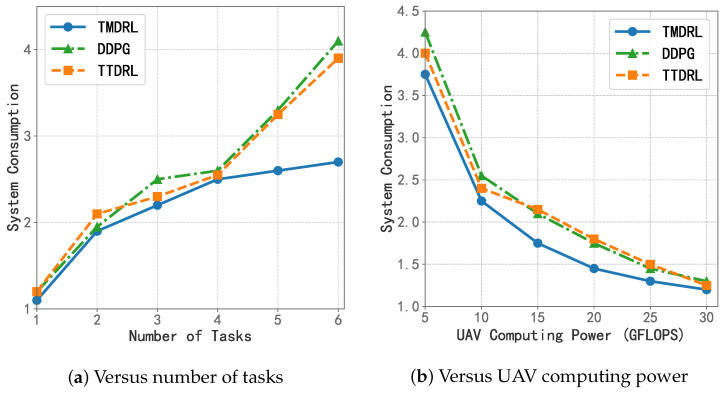
System consumption compared with the second group of algorithms.

The second set of algorithms focuses on evaluating the performance of the dual-layer multi-agent network architecture proposed. As clearly shown in Figure 8, compared with DDPG, which jointly optimizes task offloading and resource allocation, and TTDRL, which employs a unified resource allocation agent, TMDRL consistently demonstrates superior performance under varying task quantities and computing power conditions. The two baseline algorithms show mixed results, while TMDRL achieves reductions in system consumption by 12.99% and 7.82% under the conditions of *I* = 3 and $R_{\max}^{\mathrm{UAV}}$ = 10 GFLOPS, respectively. This advantage highlights the effectiveness of designing a dual-layer network structure tailored for both discrete and continuous variable optimization and of implementing multiple agents categorized by action type to collaboratively optimize task processing in scenarios involving phased execution and multiple computing entities. Such a design constitutes an adaptive and efficient enhancement over conventional reinforcement learning algorithms.

## 6. Conclusions

This paper focuses on the problem of task offloading and resource allocation in continuous task scenarios, designing a three-layer cloud edge collaboration architecture consisting of unmanned aerial vehicle networks, low orbit satellites, and ground computing centers, and proposing the two-layer based on multi-type-agent deep reinforcement learning (TMDRL) algorithm. This algorithm jointly optimizes resource allocation and task offloading while adopting improved techniques and asynchronous training strategies to increase training efficiency. Through experimental simulations, it has been proven that the algorithm proposed in this paper achieves stable optimization results under different task quantities and system resources, significantly reducing system consumption. Compared with other algorithms, the algorithm proposed in this paper has achieved the best results in both optimization performance and training cost, demonstrating significant advantages.

## Figures and Tables

**Table 1:** Communication link channel models and formulas.

Link Type	Channel Condition	Losses	Link Formula
G2A	Probabilistic line-of-sight channel	Path loss Shadowing effect	Equations (2)–(4)
A2A	line-of-sight channel	Path loss	$h^{A2A}=\frac{\beta_{0}}{{\left(d^{A2A}\right)}^{2}}$
A2S	line-of-sight channel	Path loss	$h^{A2S}=\frac{\beta_{0}}{{\left(d^{A2S}\right)}^{2}}$
S2G	line-of-sight channel	Path loss Multibeam interference	Equations (10)–(12)

**Table 2:** System model parameters.

Parameter	Value	Parameter	Value
*N*	5	$P^{A2S}$	1.5 W
*I*	1∼6	$p_{\max}^{\mathrm{LEO}}$	3 W
*H*_0_	400 m	$\sigma^{2}$	−100 dBm
*H*_1_	800 km	$G^{\max}$	30 dB
$u_{n},s_{n}$	2 km × 2 km	$G^{\mathrm{trans}}$	[10, 200] Mbit
$G^{\mathrm{cal}}$	[1, 100] GFLOPS	$Q_{\max}$	1000 Mbit
$T^{\mathrm{delay}}$	[10, 10,000] ms	*K*	3
$R_{\max}^{\mathrm{UAV}}$	[5, 30] GFLOPS	κ	10 dB
$R_{\max}^{\mathrm{LEO}}$	500 GFLOPS	$\eta_{s}$	30%
$B^{G2A}$	5 MHz	γ	1200 W/m^2^
$B^{A2A}$	5 MHz	*M*	1 m^2^
$B^{A2S}$	100 MHz	$C_{\max}$	0.8 kWh
$B^{S2G}$	100 MHz	*L*	100
$P^{G2A}$	0.2 W	$P^{A2A}$	0.5 W

## Data Availability

The data presented in this study are not publicly available due to privacy. Data are available from the corresponding author upon reasonable request.
